# The impact of organizational commitment on the professional development of primary and secondary school physical education teachers: a moderated mediation model

**DOI:** 10.3389/fspor.2026.1791333

**Published:** 2026-03-23

**Authors:** Longbiao Hu, Yanqing Zhao, Wenbo Hao, Feng Zhang

**Affiliations:** 1 Henan Sport University, Zhengzhou, China; 2 Mental Health Service Center, Yanshan University, Hebei, China; 3 College of Sports and Physical of Zhengzhou University, Zhengzhou, China; 4 Kaifeng City Bianjing Road Primary School, Kaifeng, China

**Keywords:** organizational commitment, primary and secondary school physical education teachers, school environment, teacher professional development, work engagement

## Abstract

**Introduction:**

Based on Self-Determination Theory, Educational Ecology Theory, and the Job Demands-Resources (JD-R) model, this study explores the relationship between the school environment and the professional development of primary and secondary school physical education teachers, as well as its underlying mechanisms. From an individual-school synergistic perspective, it investigates how organizational commitment influences their professional development by constructing a moderated mediation model.

**Methods:**

A sample of 526 physical education teachers was surveyed using the Work Engagement Scale, School Environment Scale, Professional Development Scale for Primary and Secondary School Teachers, and Organizational Commitment Scale.

**Results:**

The results show that organizational commitment positively predicts the professional development of primary and secondary school physical education teachers. Work engagement plays a partial mediating role in this relationship. Furthermore, both the direct path of “organizational commitment -professional development” and the first stage of the mediation path (“organizational commitment-work engagement”) are significantly moderated by the school environment.

**Discussion:**

This study proposes a theoretical pathway wherein a highly supportive school environment grants teachers instructional autonomy, provides professional resources, and fosters a climate of trust, thereby satisfying their basic psychological needs. Under such conditions, high organizational commitment is more readily internalized as self-endorsed goals, translating into more proactive and focused work engagement, which strengthens its driving effect on professional growth. When teachers experience autonomy, competence, and relatedness, they are more willing to channel their engagement into exploratory, innovative, and continuous learning behaviors, thereby effectively promoting their professional development.

## 1 Introduction

Physical education teachers serve as key agents in implementing moral education and cultivating well-rounded individuals in the new era, and thus constitute an important pillar in advancing educational modernization. In recent years, with the deepening implementation of national policies such as the “sports powerhouse” strategy, the “double reduction” policy (1), and the integration of sports and education (2), the role and value of physical education teachers have become increasingly prominent, emerging as a focal topic in contemporary educational reform and development. Against this backdrop, ecological research on teacher professional growth has continued to evolve, with growing scholarly attention to how teachers’ professional well-being and external environments shape their developmental trajectories.

A review of the existing literature in the teacher domain indicates that the field remains largely dominated by theoretical discussions, whereas rigorous empirical and quantitative evidence is still relatively limited. In particular, in-depth analyses that unpack the mechanisms linking multiple variables are notably scarce (3). Regarding research populations, prior studies have primarily focused on teachers in other subject areas (4), with insufficient attention paid to physical education teachers, especially those in primary and secondary schools.

In terms of research focus, scholars have extensively examined constructs such as teacher burnout (5), well-being (6), self-efficacy (7), and job satisfaction (8), whereas teachers' professional development has received comparatively less systematic attention. Finally, with respect to analytical perspectives, much of the literature adopts an individual-level lens, emphasizing factors such as self-efficacy (9), psychological resilience (10), and instructional engagement (11). Far fewer studies have examined teacher development through an integrated framework that explicitly captures the synergistic interplay between individual teachers and school-level organizational contexts.

Educational ecology theory offers a valuable lens for understanding the professional growth of primary and secondary school physical education teachers. It views teacher development as an ongoing interaction between individuals and their environments, shaped by both personal attributes and contextual factors (12). Thus, grounded in Educational Ecology Theory, Self-Determination Theory, and the Job Demands-Resources (JD-R) model, this study takes organizational commitment as the starting point, work engagement as the pivotal mechanism, the school environment as the contextual entry point, and the professional development of primary and secondary school physical education teachers as the ultimate outcome. From an individual-school synergistic perspective, it investigates the mechanism through which organizational commitment influences their professional development and constructs a moderated mediation model, aiming to provide a theoretical foundation for promoting the professional growth of these teachers.

## 2 Literature review

### 2.1 Direct effect of organizational commitment on professional development

Organizational commitment, as a key personal psychological resource, enhances an individual's capacity for self-regulation. According to the three-component model (13), individuals with high organizational commitment typically strongly identify with the organization's values and norms, are willing to invest more effort towards organizational goals, and demonstrate stronger work motivation. Conversely, low organizational commitment tends to lead to negative behaviors such as work disengagement (14, 15). Research indicates that organizational commitment can significantly predict a range of important work attitudes and performance outcomes, including job satisfaction and work engagement (16).

Within the teacher population, a close association exists between organizational commitment and professional development, as it influences teachers' behavioral choices and attitudinal orientations in their professional growth (49). Teachers with high organizational commitment generally hold more positive attitudes towards the education profession, engage in their work more proactively, thereby enhancing organizational efficacy, and foster their own professional growth through superior job performance (17). Consequently, Hypothesis 1 is proposed: organizational commitment has a direct effect on the professional development of primary and secondary school PE teachers.

### 2.2 The mediating role of work engagement

From a positive psychology perspective, work engagement is defined as a persistent, positive state of emotion, motivation, and cognition. For teachers, work engagement manifests as a positive, fulfilling, and enduring affective-cognitive experience exhibited during educational activities (18). Primary and secondary school PE teachers bear the mission of physical education reform and development. Ensuring their work engagement is both a prerequisite for improving teaching quality and a key factor in promoting student learning and healthy development (19). The Job Demands-Resources (JD-R) model further posits that an individual's level of work engagement increases when job resources (e.g., organizational justice) match job demands (20).

On the other hand, teachers with high work engagement are more inclined to demonstrate proactive and innovative behaviors (21), which aligns with the intrinsic requirements of teacher professional development. Further research shows that the higher a teacher's work engagement, the more they perceive meaning and value in their work, and the stronger their professional development agency becomes (22). Moreover, work engagement can function as a mediating variable, playing a transmitting role in mechanisms related to teacher development. Thus, this study proposes Hypothesis 2: work engagement mediates the relationship between organizational commitment and the professional development of primary and secondary school PE teachers.

### 2.3 The moderating role of the school environment

According to Conservation of Resources Theory, individuals are motivated to maintain and acquire resources, a tendency which drives their work behavior (23). Organizational commitment, as a crucial personal psychological resource, enhances teachers' capacity for self-regulation. The school environment, as an external contextual factor, has the potential to interact with the individual internal factor of organizational commitment. Therefore, it is necessary to examine whether this interaction moderates the underlying mechanism, specifically, whether the school environment moderates the path from organizational commitment to work engagement.

Although organizational commitment has direct or indirect effects on the professional development of primary and secondary school PE teachers, this process may be moderated by the external environment. Research indicates that when the environment satisfies teachers' basic psychological needs, it helps to enhance their self-efficacy and work efficiency (24), thereby promoting professional growth. Consequently, advancing teacher professional development requires an integrated approach that addresses both individual internal factors and external environmental support. Based on this, the present study proposes Hypothesis 3: the school environment moderates the mediated pathway from organizational commitment to the professional development of primary and secondary school PE teachers through work engagement.

**Figure 1:**
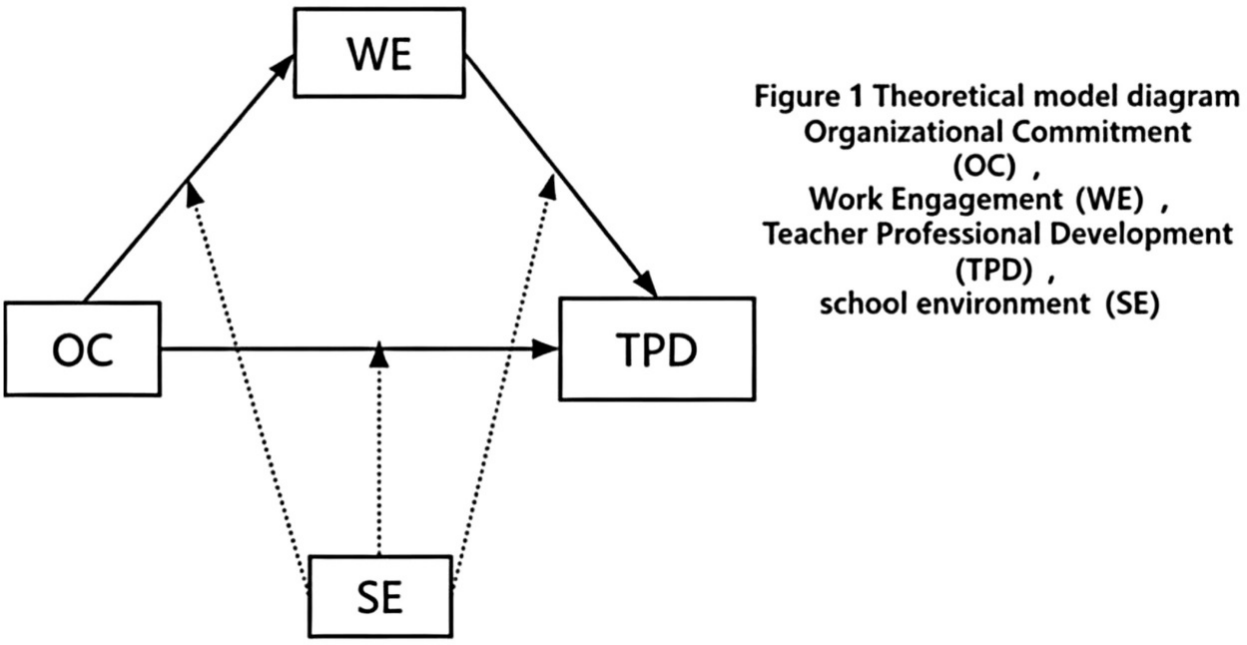
Theoretical model diagram.

In summary, this study focuses on how organizational commitment promotes the professional development of primary and secondary school PE teachers. By integrating school-level factors (the objective school environment) and individual-level factors (work engagement), a moderated mediation theoretical model is constructed (see Figure 1). The specific research hypotheses are as follows: (1) organizational commitment positively predicts the professional development of primary and secondary school PE teachers; (2) work engagement mediates the relationship between organizational commitment and their professional development; (3) the school environment moderates the relationships between organizational commitment and work engagement, as well as between organizational commitment and teacher professional development. The school environment has a moderating effect in the first half and the second half of the mediating path and the direct path.

## 3 Methods

### 3.1 Participants

Following standard principles of questionnaire design, this study employed a combination of convenience sampling and cluster sampling to recruit physical education (PE) teachers from primary and secondary schools in Henan Province. A total of 580 questionnaires were administered. After excluding invalid questionnaires due to incomplete responses, 526 valid questionnaires remained, yielding an effective response rate of 90.69%.A sensitivity analysis was performed using G*Power 3.2.9.2. The results indicated that, with a significance level of （*α*= 0.05） and a statistical power of （0.80）, the current sample size was sufficient to detect even small effect sizes (*f* = 0.12), demonstrating the adequacy of the sample size for this study (25).

regarding the demographic characteristics, the sample consisted of 283 male PE teachers (53.8%) and 243 female PE teachers (46.2%). In terms of age, 186 participants were aged 30 or below (35.4%), 169 were between 31 and 40 years old (32.1%), 125 were between 41 and 50 years old (23.8%), and 46 were aged 51 or older (8.7%).To assess potential non-response bias, the valid sample was divided into two groups based on the chronological order of retrieval, following the extrapolation method proposed by Armstrong and Overton (26). The first 30% of returned questionnaires were defined as the “early response group” (*n* = 158), and the last 30% were defined as the “late response group” (*n* = 158). Subsequently, Chi-square tests were performed to compare these groups across key demographic variables, specifically gender and age. The results indicated no statistically significant differences between early and late respondents in terms of gender distribution (*χ^2^* = 1.24, *p* = 0.265 > 0.05) or age distribution (*χ^2^*= 3.58, *p* = 0.167 > 0.05). These findings suggest that serious non-response bias was not present in the data, indicating that the sample possesses acceptable representativeness.

### 3.2 Instruments

#### 3.2.1 School environment scale

Based on the “Classification Methods of School Factors” developed by Hoy and Woolfolk, and adapted to the actual conditions of China and the characteristics of school teaching environments, the “School Environment” scale was employed (27). This scale consists of 19 items across five dimensions: Principal Influence, Job-Provided Development Conditions, School Atmosphere, Interpersonal Relationships, and Physical Environment. Responses are scored on a 5-point Likert scale ranging from 1 (“Completely Disagree”) to 5 (“Completely Agree”). In this study, the scale demonstrated good internal consistency with a Cronbach's alpha of 0.879. Confirmatory factor analysis indicated an acceptable model fit: *χ*²/df = 1.745, RMSEA = 0.036, NFI = 0.917, RFI = 0.921, IFI = 0.916, TLI = 0.952, CFI = 0.924.

#### 3.2.2 Professional development scale for primary and secondary school teachers

The Professional Development Scale for Primary and Secondary School Teachers, developed by Chen et al. (28), comprises 21 items. It measures five dimensions: Professional Affection, Professional Development Actions, Professionalization Beliefs, Professional Problem-Solving, and Professional Knowledge and Skills. Responses are recorded on a 5-point Likert scale from 1 (“Strongly Disagree”) to 5 (“Strongly Agree”), with higher scores indicating a higher level of individual professional development. In the present study, the scale showed good internal consistency (*α*= 0.885). Confirmatory factor analysis results were acceptable: *χ*²/df = 2.411, RMSEA = 0.052, NFI = 0.901, RFI = 0.877, IFI = 0.913, TLI = 0.902, CFI = 0.942.

#### 3.2.3 Work engagement scale

The Work Engagement Scale was used in this study as cited in Schaufeli et al. (18). It contains 17 items rated on a 5-point frequency scale from 1 (“Almost Never”) to 5 (“Always”). Higher scores reflect a greater degree of individual work engagement. The scale exhibited excellent internal consistency in this study (*α*= 0.972). Confirmatory factor analysis indicated a good model fit: *χ*²/df = 2.098, RMSEA = 0.045, NFI = 0.922, RFI = 0.953, IFI = 0.972, TLI = 0.923, CFI = 0.963.

#### 3.2.4 Organizational commitment scale

The three-dimensional Organizational Commitment Scale developed by Allen and Meyer (29) served as the theoretical basis for measurement. Its applicability in teacher research has been demonstrated and widely recognized by scholars both domestically and internationally (30). The scale consists of 14 items rated on a 5-point Likert scale from 1 (“Strongly Disagree”) to 5 (“Strongly Agree”), with higher scores indicating a higher level of organizational commitment. Confirmatory factor analysis yielded acceptable fit indices: *χ*²/df = 4.37, RMSEA = 0.08, NFI = 0.933, RFI = 0.927, IFI = 0.935, TLI = 0.918, CFI = 0.947, indicating good structural validity of the scale in this study. The overall internal consistency coefficient for the scale was 0.873.

### 3.3 Data analysis

The data were organized and analyzed using SPSS 27.0 and AMOS 24.0 software. The statistical methods employed included reliability and validity tests, common method bias test, descriptive statistics and correlation analysis of the variables. Moderated mediation effect analysis was conducted using the bootstrapping method. Furthermore, given that this study was designed as an observational survey, to ensure transparency and completeness in methodological procedures and result reporting, the study was conducted in accordance with the standardized reporting guidelines proposed by Sanchez et al. for enhancing the quality of research reports (31).

## 4 Results

### 4.1 Common method bias test

To control for the potential influence of common method bias, measures such as anonymous responses and reverse-scoring were implemented during the survey. Nevertheless, as the questionnaire relied on self-reported data, common method bias may still exist. Harman's single-factor test was employed to assess common method bias. The results indicated that there were eight factors with eigenvalues greater than 1, and the first factor accounted for 29.95% of the variance (less than 50%) (32). Additionally, a confirmatory factor analysis was conducted to test a single-factor model (33). The results showed poor model fit indices (*χ*²/df = 10.23, RMSEA = 0.118, NFI = 0.427, RFI = 0.411, IFI = 0.452, TLI = 0.451, CFI = 0.454). Therefore, no severe common method bias was present in this study.

### 4.2 Descriptive statistics and correlation analysis of variables

Descriptive statistics and the correlation matrix for all variables are presented in Table 1. The results of SPSS correlation analysis revealed that organizational commitment, work engagement, teacher professional development, and the school environment were all significantly positively correlated with each other (*p* < 0.001). This finding supports the notion that the professional development of primary and secondary school physical education teachers is influenced by multiple factors. Furthermore, age and gender showed no significant correlations with any of the core study variables (school environment, professional development, work engagement, organizational commitment). This indicates that, within the current sample, the professional psychological states and behavioral expressions of the physical education teachers were not significantly influenced by their age or gender.

**Table 1:** Descriptive statistics and correlation analysis of variables (*n* = 526).

Variables	1	2	3	4	5	6
SE	-					
TPD	0.634***	-				
WE	0.613***	0.604***	-			
OC	0.197***	0.321***	0.271***	-		
Age	−0.049	0.000	0.009	0.420	-	
Sex	0.041	−0.024	−0.012	−0.022	−0.108	-
M	2.06	1.46	3.55	3.53	3.85	3.12
SD	0.97	0.50	0.69	0.60	0.91	0.56

*** indicate significance at 1%. Organizational commitment (OC), Work engagement (WE), Teacher professional development (TPD), School environment (SE).

### 4.3 Mediation effect test

The mediating effect of work engagement was examined using Model 4 of the PROCESS macro for SPSS (34). A bootstrapping procedure with 5,000 resamples was performed to estimate the 95% confidence intervals (see Tables 2, 3). Prior to the analysis, all continuous variables—specifically organizational commitment, school environment, and work engagement—were z-standardized to facilitate the interpretation of effects and mitigate potential multicollinearity. The Variance Inflation Factors (VIF) for all predictor variables were below 2.07, indicating that multicollinearity was not a serious concern in the current model. Initial results indicated that organizational commitment had a significant positive predictive effect on the professional development of primary and secondary school PE teachers (*β* = 0.344, t = 8.273, *p* < 0.001), thus supporting Hypothesis 1. In Equations 2 and 3, after introducing work engagement as the mediating variable, the positive predictive effect of organizational commitment on professional development remained significant (*β* = 0.282, t = 4.792, *p* < .001). Concurrently, the effects of organizational commitment on work engagement (*β* = 0.438, t = 6.433, *p* < 0.001) and of work engagement on professional development (*β* = 0.365, t = 15.761, *p* < 0.001) were both statistically significant.

**Table 2:** Mediation effect tests.

Variables
	Dependent Variable:（PETPD）			Dependent Variable:（WE）			Dependent Variable:（PETPD）		
	*β*	*t*	*95%CI*	*β*	*t*	*95%CI*	*β*	*t*	*95%CI*
Age	0.022	1.134	[−0.017, 0.062]	0.035	1.025	[−0.025, 0.087]	0.025	0.783	[−0.021, 0.062]
Sex	−0.060	−1.437	[−0.136, 0.097]	−0.021	−0.410	[−0.151, 0.089]	−0.043	−1.329	[−0.123, 0.029]
OC	0.344	8.273***	[0.260, 0.432]	0.438	6.433***	[0.308, 0.579]	0.182	4.792***	[0.108, 0.257]
WE							0.365	15.761***	[0.320, 0.411]
R	0.321			0.271			0.626		
R²	0.103			0.632			0.392		
F	60.104***			41.389***			168.443***		

*** indicate significance at 1%. Organizational commitment (OC), Work engagement (WE), Teacher professional development (TPD), School environment (SE).

**Table 3:** Decomposition of total, direct, and indirect effects.

Effect type	Effect	Boot SE	BootLLCI	BootULCI	Proportion of Total Effect
Direct Effect	0.182	0.038	0.108	0.257	52.91%
Indirect Effect	0.162	0.032	0.101	0.227	47.09%
Total Effect	0.344	0.044	0.260	0.432	

Bootstrap analysis (5,000 samples) showed that the direct effect of organizational commitment was 0.182 [SE = 0.038, 95% CI (0.108, 0.257)], accounting for 52.91% of the total effect. The indirect effect was 0.162 [SE = 0.032, 95% CI (0.101, 0.227)], accounting for 47.09% of the total effect. As none of the confidence intervals contained zero, the results indicate that work engagement plays a partial mediating role in the relationship between organizational commitment and professional development, Notably, indirect effects account for a striking 47.09% of the total impact, indicating that nearly half of organizational commitment's effectiveness in advancing physical education teachers' professional development must be channeled through the pathway of motivating work engagement, thereby supporting Hypothesis 2 (35).

### 4.4 Moderated mediation model

**Figure 2:**
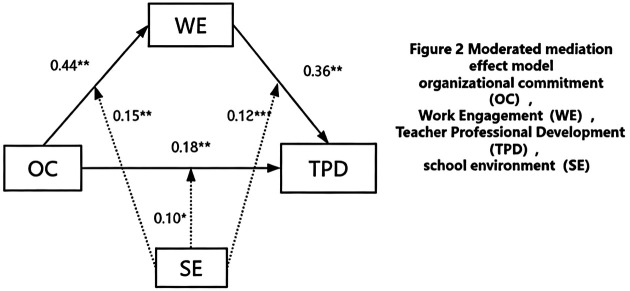
Moderated mediation effect model.

The moderated mediation model proposed in this study was tested following the analytical procedures outlined by Wen (36). To mitigate potential multicollinearity between the interaction terms and their corresponding main effects, all continuous variables (i.e., organizational commitment, school environment, and work engagement) were z-standardized prior to the construction of the product terms. The Variance Inflation Factors (VIF) for all predictor variables ranged from 1.02 to 2.069, indicating that multicollinearity was not a serious concern in the current study. Subsequently, a moderated mediation analysis was performed using Model 14 of the PROCESS macro for SPSS (37), with gender and age included as covariates. The parameter estimates for each regression equation are presented in Table 4. The results demonstrated that organizational commitment significantly predicted the professional development of primary and secondary school PE teachers (*β* = 0.21, t = 4.02, *p* < 0.001), as well as work engagement (*β* = 0.44, t = 4.01, *p* < 0.01). Furthermore, the interaction effect between organizational commitment and school environment was significant (*β* = 0.15, t = 3.06, *p* < 0.01), indicating that the school environment moderates the first stage of the mediation path. When predicting professional development with all variables included, significant interaction effects emerged for both organizational commitment and school environment (*β* = 0.10, t = 2.01, *p* < 0.05) and work engagement and school environment (*β* = 0.12, t = 3.37, *p* < 0.001). These results indicate that the school environment continues to moderate both the direct path and the second stage of the mediation path, thereby supporting Hypothesis 3. Additionally, work engagement effectively predicted professional development (*β* = 0.36, t = 4.42, *p* < 0.001), while the predictive effect of organizational commitment on professional development remained significant (*β* = 0.18, t = 5.40, *p* < 0.001). This confirms that work engagement plays a partial mediating role in the relationship between organizational commitment and professional development, supporting Hypothesis 2. Overall, a favorable school environment systematically strengthens the complete “Organizational Commitment, Work Engagement, Professional Development” mechanism. Specifically, in a highly supportive environment, the driving effect of organizational commitment on work engagement is stronger (first-stage moderation), the conversion efficiency of work engagement into professional development is higher (second-stage moderation), and the direct impact of organizational commitment on professional development is more pronounced (direct path moderation), ultimately forming a positive cycle (see Figure 2).

**Table 4:** Moderated mediation results.

Variables	Dependent Variable:（PETPD）			Dependent Variable:（WE）			Dependent Variable:（PETPD）		
	*β*	*t*	*95%CI*	*β*	*t*	*95%CI*	*β*	*t*	*95%CI*
Age	0.018	1.01	[−0.017, 0.058]	0.043	1.487	[−0.013, 0.102]	0.013	0.670	[−0.024, 0.048]
Sex	−0.046	−1.262	[−0.012, 0.026]	0.018	0.291	[−0.095, 0.128]	−0.052	−1.439	[−0.122, 0.019]
OC	0.21	4.02***	[0.01, 0.60]	0.44	4.01**	[0.11, 0.67]	0.18	5.40***	[0.13, 0.28]
SE	0.49	8.01***	[0.38, 0.62]	0.59	7.50***	[0.35, 0.80]	0.31	5.51***	[0.33, 0.67]
OC × SE	0.12	2.48*	[0.04, 0.13]	0.15	3.06**	[0.05, 0.25]	0.10	2.01*	[0.02, 0.18]
WE							0.36	4.42***	[0.40, 0.65]
WE × SE							0.12	3.17***	[0.07, 0.17]
*R²*	0.40			0.45			0.55		
*F*	78.25***			129.50***			125.20***		

*, **, and *** indicate significance at 10%, 5%, and 1%, respectively. respectively. Organizational commitment (OC), Work engagement (WE), Teacher professional development (TPD), School environment (SE), Organizational commitment×School environment (OC × SE), Work engagement ×School environment (WE × SE).

## 5 Discussion

### 5.1 The direct effect

Organizational commitment exerts a direct positive influence on the professional development of primary and secondary school physical education (PE) teachers. Specifically, PE teachers with higher organizational commitment benefit more strongly from the positive effect of work engagement on their professional growth. This study further reveals that the school environment moderates the impact of organizational commitment on teachers' development, and this moderating effect spans the entire mediation process. In other words, the strength of the relationship between organizational commitment and work engagement is contingent on the school environment.

From the theoretical perspective of the three-factor structure model (38) and the organizational matching model (39), organizational commitment is best understood not as a singular attitude or behavior, but as a multidimensional construct integrating cognitive, affective, and behavioral dimensions. It reflects a teacher's identification with and emotional attachment to the school, as well as a behavioral tendency to remain with the organization based on professional interests and positional considerations. In practice, PE teachers with high organizational commitment generally exhibit more positive educational beliefs and value perceptions, which in turn stimulate stronger work motivation and enhance organizational effectiveness. They are also more likely to engage proactively in teaching, reduce unproductive work behaviors, and drive their own professional advancement through consistent high-quality performance.

Enhancing organizational commitment among primary and secondary school PE teachers is thus a crucial pathway for supporting their professional development. Given the diversity in developmental characteristics and learning needs within this teacher population, a variety of support mechanisms is necessary. Schools should actively cultivate a coherent organizational climate, establish conditions favorable to PE teachers' growth, and prioritize the stimulation and fulfillment of their achievement needs (40). Since the school environment and cultural atmosphere significantly shape teachers' organizational commitment (41), strengthening organizational culture can help teachers build and sustain identification with and dedication to the school. Accordingly, PE teachers should consciously align their personal career objectives with the school's developmental vision to prevent major value misalignment and maintain a strong sense of organizational belonging. Finally, from the perspective of teacher agency, it is essential to reinforce PE teachers' sense of professional autonomy, instill a lifelong learning mindset, set clear developmental goals, and continually unlock potential through ongoing learning and innovative practice. By advancing their professional competence while deepening organizational commitment, a virtuous cycle of interactive growth can be achieved.

### 5.2 The mediating effect

This study confirms the mediating role of work engagement in the relationship between organizational commitment and the professional development of primary and secondary school PE teachers. The results indicate that work engagement has a significant impact on teacher professional development. Organizational commitment not only directly and positively predicts professional development but also exerts an indirect promoting effect through the mediating pathway of work engagement. As a key mediating variable, work engagement serves as a connecting and transmitting link between organizational commitment (the independent variable) and the professional development of PE teachers (the dependent variable).

Drawing upon the Job Demands-Resources (JD-R) model, this study elucidates the mechanism through which work engagement mediates the relationship between organizational commitment and teacher professional development. The JD-R model posits that working conditions instigate two independent processes: the health impairment process (energy consumption) and the motivational process. Within this framework, job resources stimulate intrinsic motivation and foster positive psychological states, thereby enhancing work engagement and job performance (42). Sustaining work engagement necessitates the support of job resources; as a critical resource, organizational commitment not only facilitates goal attainment but also buffers the detrimental effects of job demands on performance and well-being (43). Conversely, prolonged exposure to a resource-depleted environment predisposes teachers to emotional exhaustion and burnout (44). For primary and secondary school physical education (PE) teachers, enhancing work engagement bolsters vigor and perceived competence while contributing to workforce stability (45), which subsequently drives professional growth. Consequently, when school organizations provide ample developmental resources and supportive conditions, they directly augment teachers' job resources. This augmentation elevates work engagement levels, ultimately fostering professional development through superior job performance.

In summary, organizational commitment can indirectly foster the professional development of PE teachers through work engagement. Thus, enhancing teacher work engagement represents a critical lever for promoting professional growth. This is especially pertinent for newly recruited PE teachers, who often expend considerable job resources on non-instructional tasks during role adaptation, relationship-building, and organizational socialization, thereby reducing the time and energy available for professional development. Strengthening their work engagement during this crucial phase can facilitate a smoother transition and accelerate professional growth. To achieve this, schools should implement structured pre-service training that helps new teachers adapt quickly to their roles and equips them with skills to tailor physical education instruction to adolescents' physical and psychological characteristics, enabling more effective engagement. Moreover, schools should allocate additional job resources to PE teachers, who typically carry a heavy workload that extends beyond classroom teaching to include morning exercises, recess activities, and other duties. Increasing their job resources helps preserve teaching energy and maintain work quality. Finally, fostering a positive work environment is essential. A supportive organizational climate provides teachers with positive affective experiences, which in turn stimulates work motivation, enhances self-efficacy, raises work engagement and efficiency, and ultimately promotes the professional development of PE teachers.

### 5.3 The moderating effect

This study found that the professional development of primary and secondary school PE teachers is influenced by the school environment. Furthermore, it validates the hypothesis that the school environment exerts a moderating effect within the relationship between organizational commitment, work engagement, and professional development.

From the perspective of educational ecology theory, the school environment provides the arena for the exchange of energy and materials necessary for teacher professional development. Positive, dynamic interactions within this arena help stabilize the ecological structure and maintain systemic balance, thereby supporting the sustainable development of teachers (12). Furthermore, from the perspective of Self-Determination Theory (SDT), the interaction between the environment and the individual can be explained by emphasizing the agentic role of the self in the motivational process. The theory posits that human self-determination lies in the flexible regulation of interactions between oneself and the environment, while also underscoring the critical influence of the environment on the realization of human potential. A highly supportive school environment grants teacher's instructional autonomy, provides professional development resources, and fosters a climate of trust and collaboration, thereby satisfying teachers' basic psychological needs. Under such conditions, high organizational commitment is more readily internalized as self-endorsed goals, which in turn translates into more proactive, focused, and vigorous work engagement. This further amplifies the driving effect of work engagement on professional growth. When teachers experience autonomy, competence, and relatedness, they are more willing to channel their engagement into exploratory, innovative, and continuous learning behaviors, thereby promoting their professional development (46).As a crucial context for teachers' affective experiences and professional behaviors, the school environment has been empirically verified to enhance teachers' sense of success and professional confidence, alleviate occupational stress, and foster the development of positive psychological qualities and growth trajectories (47). In summary, as a key component of the educational ecosystem, the school environment not only plays a vital supportive role in teacher growth but also provides an essential platform for knowledge acquisition, work execution, and self-expression (48). Therefore, cultivating a school environment characterized by an excellent culture, harmonious interpersonal relationships, and adequate physical conditions is paramount for safeguarding and advancing the professional development of primary and secondary school PE teachers.

## 6 Conclusion

Primary and secondary school physical education teachers should consciously align their personal career goals with the school's developmental vision to prevent significant misalignment in values, thereby sustaining a strong sense of organizational belonging. Moreover, teachers should enhance their sense of professional agency, adopt a lifelong learning mindset, set clear developmental objectives, and continuously unlock their potential through ongoing learning and innovative practice. By advancing personal professional competence while simultaneously deepening organizational commitment, a virtuous cycle of mutual development can be established.

## 7 Limitations

This study has several limitations. First, in terms of research design, the data were collected through a cross-sectional survey. Although a moderated mediation model was employed to clarify relationships among variables, the longitudinal stability and causal direction of these relationships require further verification. Future studies could adopt longitudinal or experimental designs to examine the temporal dynamics and causal pathways among variables. Second, regarding the constructs measured, organizational commitment, professional development, school environment, and work engagement are all multidimensional. Distinct mechanisms may operate across different dimensions. Subsequent research should therefore conduct finer-grained analyses to disentangle the specific influence pathways between sub-dimensions. Finally, methodologically, the present study relied mainly on quantitative analysis. Future work could integrate qualitative approaches, such as interviews, or experimental methods within a mixed-methods design, to gain a more comprehensive and nuanced understanding of the processes and mechanisms driving physical education teachers' professional development.

## Data Availability

The original contributions presented in the study are included in the article/Supplementary Material, further inquiries can be directed to the corresponding author.
